# Supplementary material for: Traditional Chinese Medicine for Topical Treatment of Skeletal Muscle Injury
Source: Pharmaceuticals (Basel). 2023 Aug 12;16(8):1144. doi: 10.3390/ph16081144 (PMC10457816; doi:10.3390/ph16081144)
Supplement: Supplementary file 1 [file pharmaceuticals-16-01144-s001.zip › pharmaceuticals-2504739-supplementary.pdf]

Supplementary Materials

Table S1. Chemical composition of CDR paste.

Herb	Chemical marker	Content (mg/g) of paste
Carthami Flos (C)	Hydroxysafflor yellow A (HYA)	2.14
	Kaempferol (KAE)	0.004
Dipsaci Radix (D)	Asperosaponin VI (ASP)	19.23
	Oleanolic acid (OA)	0.05
Rhei Rhizoma (R)	Emodin (EMO)	0.96
	Rhein (RHE)	1.00

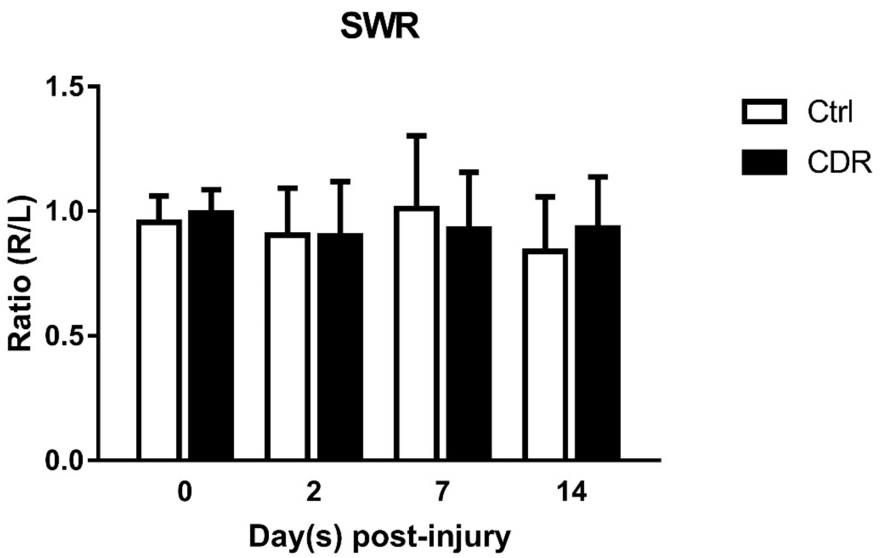

Figure S1. The static weight ratio (SWR) of the rats throughout the experiment. R and L mean the right (contused) leg and left (normal leg without contusion) leg, respectively. Data ~~is~~ are presented as mean and the error bars indicate the standard deviation.
